# Post metabolic bariatric surgery weight regain: the importance of GLP-1 levels

**DOI:** 10.1038/s41366-024-01461-2

**Published:** 2024-01-15

**Authors:** Nursel Çalık Başaran, Idit Dotan, Dror Dicker

**Affiliations:** 1 Hacettepe University, Faculty of Medicine, Department of Internal Medicine, General Internal Medicine, Ankara, Türkiye; 2 Rabin Medical Center, Beilinson Hospital, Department of Endocrinology and Obesity Clinic, Petah Tikva, Israel; 3 Tel Aviv University, Faculty of Medicine, Tel Aviv, Israel; 4 Rabin Medical Center, Hasharon Hospital, Department of Internal Medicine and Obesity Clinic, Petah Tikva, Israel

**Keywords:** Obesity, Bariatric surgery, Weight management

## Abstract

Weight regain and insufficient weight loss are essential problems after metabolic bariatric surgery (MBS) in people living with obesity. Changes in the level of glucagon-like peptide-1 (GLP-1) secreted from the gut after bariatric surgery are one of the underlying mechanisms for successful initial weight loss. Studies and meta-analyses have revealed that postprandial GLP-1 levels increase after the Roux-en-Y gastric bypass and sleeve gastrectomy, but fasting GLP-1 levels do not increase significantly. Some observational studies have shown the relationship between higher postprandial GLP-1 levels and successful weight loss after bariatric surgery. There is growing evidence that GLP-1-receptor agonist (GLP-1-RA) use in patients who regained weight after bariatric surgery has resulted in significant weight loss. In this review, we aimed to summarize the changes in endogenous GLP-1 levels and their association with weight loss after MBS, describe the effects of GLP-1-RA use on weight loss after MBS, and emphasize metabolic adaptations in light of the recent literature. We hypothesized that maintaining higher basal-bolus GLP-1-RA levels may be a promising treatment choice in people with obesity who failed to lose weight after bariatric surgery.

## Introduction

Metabolic Bariatric surgery (MBS) is an effective long-term treatment for obesity when combined with lifestyle modification. Nevertheless, failures to achieve maximal weight loss and maintain the weight loss achievement have been described. A failure can be defined as weight regain (WR) after successful initial weight loss or insufficient weight loss (IWL). Excess weight loss (EWL) of at least 50% and remission of comorbidities are considered a success post MBS. Although no specific definition is used in the literature for clinically significant WR, it can be defined as progressive weight gain after a successful initial weight loss [1]. IWL can be defined as less than 50% EWL at 18 months post MBS [1].

The occurrence and magnitude of WR after MBS differs in the literature according to the type of surgery, definition of WR, length of the follow-up period, and follow-up rates. In the Longitudinal Assessment of Bariatric Surgery (LABS) study by Courcoulas et al. [2], the median time to reach nadir weight loss was 2 years after a Roux-en-Y gastric bypass (RYGB). Five years after reaching the nadir of weight loss, 50.2% of the RYGB patients regained more than 15% of maximal weight loss and 86.5% regained more than 10% of maximal weight loss [3]. Cooper et al. [4] reported that the mean WR was 23.4% of maximal weight loss at 7 years after RYGB and that 37% of patients regained more than 25% of their total weight loss. They also observed that participants who lost more weight in the first year after surgery had a greater absolute weight loss over time; however, WRs did not differ significantly in relationship to first-year weight loss rates [4]. In the systematic review by Lauti et al. [5], a high heterogeneity of WR rates after sleeve gastrectomy (SG) was reported and accordingly, 5.7–20% of the patients had significant WR at 2 years post SG, while 26.3–76% of them had significant WR at 6 years post SG. There is no standard definition for WR in the literature; however, most of the studies accept the definition of WR as 10 kg of weight gain after reaching nadir weight. In another systematic review, Clapp et al. [6] estimated the recidivism rate as 27.8% (ranging from 14 to 37%) at 7 years or more post SG. In their systematic review, Athanasiadis et al. [7] reported that 17.6% of the patients had a WR higher than 10% after the primary MBS (SG and RYGB) in a mean follow-up duration of 62 months; this can be summarized as 1 of 6 patients experiencing a WR of 10% or more post MBS. There is insufficient data on IWL after primary MBS, but studies have shown that 32–40% of the revisional surgeries are due to IWL after primary MBS [8, 9].

### Glucagon-like peptide-1 (GLP-1) Levels after MBS

Besides dietary nonadherence and physical inactivity, psychiatric, anatomical, genetic, and hormonal causes are suggested to be underlying reasons for WR or IWL post MBS [1, 7]. In normal-weight individuals, GLP-1 levels are very low after overnight fasting, increase rapidly with food intake, and do not return to the morning fasting level between meals [10]. Both fasting and post-prandial GLP-1 levels are lower in people with obesity as compared with those in normal-weight subjects, as obesity is characterized by a blunted post-prandial GLP-1 increase [11]. After MBS, especially after SG and RYGB, GLP-1 and peptide YY (PYY) levels increase, ghrelin levels decrease, and the peak rise (at 15–30 min) in GLP-1 levels after mixed meal test or oral glucose tolerance test (OGTT) is higher than before BMS [12, 13].

In their study, Bose et al. [14] compared GLP-1 levels in patients after RYGB and gastric banding and showed a significant increase in the post-prandial GLP-1 levels after RYGB, but not after gastric banding. They observed that the rise in GLP-1 levels was more pronounced after 1 year than at the time point of the 12 kg weight loss. Fasting GLP-1 levels were similar after both surgeries [14]. Similarly, in a meta-analysis by Jirapinyo et al. [15], the post-prandial GLP-1 levels significantly increased, but the fasting GLP-1 levels did not increase during the 1–12 months after RYGB. Other studies show that the increase in post-prandial GLP-1 after SG lasts for at least 1 year and is comparable to biliopancreatic diversion (BPD) and RYGB [16–19]. Post-prandial GLP-1 increase is observed as early as 3 days after MBS and is more pronounced after RYGB than after SG [20, 21]. Magro et al. [22] compared glucose metabolism parameters and post-prandial GLP-1 levels before and after MBS between patients with active or inactive Crohn’s Disease and healthy controls. They showed that while the fasting and post-prandial GLP-1 levels in the pre-RYGB surgery patients were lower than in lean controls, the post-prandial GLP-1 levels became higher post-RYGB, whereas fasting GLP-1 levels did not [22].

Min et al. [23] followed the changes in GLP-1 levels of patients during OGTT pre-operatively at 1 month, 6 months, and 4–7 years after laparoscopic SG and BPD. They showed significant increases in the post-prandial GLP-1 levels at 1 and 6 months post SG, but this was not maintained at 4 years; the fasting and post-prandial GLP-1 levels decreased to an even lower value from baseline levels at 4–7 years post SG [23]. However, in the BPD group, there were significant increases in both fasting and post-prandial GLP-1 levels even after 7 years post-surgery [23]. These studies show the post-prandial increase in GLP-1 levels post MBS, without an increase in fasting GLP-1 levels. The mechanism of post-prandial increase in GLP-1 level following MBS can be explained by faster gastric emptying and rapid delivery of nutrients to the intestine, exclusion of foregut, and changes in bile acids metabolism [18, 24]. The critical question is whether there is an association between the level to which GLP-1 increases and the period of that increment and extent of weight loss post MBS.

There are studies showing that patients who experience more robust weight loss after MBS have higher levels of GLP-1 as compared with patients who have less favorable weight loss [12, 13, 24–28]. le Roux et al. [13] evaluated the post-prandial gastrointestinal hormone levels in patients with robust weight loss (>30% of total body weight) and those with poor weight loss (<25% of total body weight) at 25.3 months after RYGB. They found that the post-prandial PYY and GLP-1 responses were attenuated in patients with poor weight loss as compared to those with robust weight loss. They also reported that inhibiting GLP-1 and PYY responses by a somatostatin analog (octreotide) increased appetite and food intake [13]. Similarly, in their cross-sectional study, Dirksen et al. [27] also evaluated the fasting and post-prandial gastrointestinal hormone levels in patients with robust weight loss (≥60% EWL) and poor weight loss (≤50% of EWL) at 18.9 months post RYGB. Patients with robust weight loss had a more significant release of GLP-1 and a greater suppression of ghrelin during a mixed-meal test as compared to those with poor weight loss; however, the PYY response was similar in both patient groups. On the other hand, fasting ghrelin, GLP-1, and PYY levels did not differ between the two patient groups [27].

Santo et al. [25] compared the gut hormone level differences between patients who had successful and durable weight loss after MBS and patients who could not sustain the initial weight loss and regained more than 50% of their weight loss 4.9 years after RYGB. They observed that the fasting GLP-1 levels did not differ between the groups; however, as compared to patients with WR, there was a significantly higher increase in the post-prandial GLP-1 levels in patients who sustained the initial weight loss [25]. In another cross-sectional study of 34 patients, Shantavasinkul et al. [29] showed that patients with sustained weight loss had significantly higher post-prandial GLP-1 levels than patients with WR at 5 years post RYGB, with similar fasting GLP-1 levels. They also compared “hunger” and “desire to eat savory food or sweets” during a mixed-meal test. The post-prandial GLP-1 levels and the areas under the curve of GLP-1 were positively correlated with satisfaction and fullness, which implied that post-prandial GLP-1 release was related to appetite control in the patients who underwent RYGB, similar to individuals that did not undergo MBS [29]. Furthermore, Nannipierri et al. [18] found that patients in remission of type 2 diabetes mellitus (T2DM) after MBS (RYGB and SG) had higher fasting GLP-1 levels as compared to patients not in remission. The post-prandial GLP-1 levels significantly increased at 15 days and 1 year after MBS and this increase was pronounced in the early period after surgery in both remitters and non-remitters [18].

In their prospective study, Arakawa et al. [30] monitored the changes in gastrointestinal hormones at 1 year after RYGB and SG. They obtained a significant sustainable increase in the post-prandial GLP-1 levels as compared with the pre-surgical levels after RYGB. Post-prandial GLP-1 levels also increased after SG (though less than the increase after RYGB), but the increase was not sustainable at 1 year post SG. These changes were in parallel with the decrease in hunger and increase in satiety post MBS [30].

Besides higher GLP-1 levels, an intact hypothalamic-gut axis is essential to increase satiety and decrease hunger. Dischinger et al. [31] showed the importance of this axis in a prospective study of patients with hypothalamic obesity. Comparing the non-operated patients with hypothalamic obesity with patients having obesity (who underwent MBS or not) and lean controls, a significant increase was observed in the post-prandial GLP-1 levels in patients with hypothalamic obesity who underwent MBS [31]. However, despite these higher GLP-1 levels, hunger rates were higher and satiety was lower in the hypothalamic obesity group in comparison to other groups [31]. This study was also important in showing that fasting GLP-1 levels of patients with obesity were lower than those of lean controls (regardless whether they underwent MBS or not). After MBS, the increase in post-prandial GLP-1 levels was comparable with lean controls, whereas the fasting GLP-1 levels remained lower than in lean controls [31]. On the other hand, Sima et al. [32] evaluated gastrointestinal hormone responses to OGTT and fasting levels in patients with robust weight loss (≥50% EWL) and poor weight loss (<50% EWL) at 11.7 years post RYGB. They found no differences in the fasting and post-prandial GLP-1 and PYY levels between the two groups [32]. Additionally, Lampropoulos et al. [33] showed that the post-prandial GLP-1 response did not significantly differ between patients who maintained and regained weight at 7 years or more post MBS. These two studies differ from other studies showing associations of higher post-prandial GLP-1 levels in patients having robust weight loss and sustained weight loss. There are likely other mechanisms for long-term weight loss and weight maintenance after MBS and further investigation would be in order.

### Effects of GLP-1-receptor agonists (GLP-1-RA) in patients after MBS

Revisional surgeries have been the main option for the treatment of MBS failure up to recent years; however, anti-obesity drugs are becoming a promising treatment option as we understand the gastrointestinal hormonal changes and their association with weight loss post MBS. This raises the question whether GLP-1-RAs are effective in patients who fail to lose a significant amount of weight or regain weight post MBS. A summary of the studies in the literature regarding GLP-1-RAs use after MBS is given in Table 1.

**Table 1:** Summary of studies in the literature evaluating glucagon-like peptide-1-receptor agonist use in patients who regained weight or lost insufficient weight after metabolic bariatric surgery.

Studies	Study design	Number of subjects	Type of surgery	Inclusion criteria	Time since the operation	Type of GLP-1-RA	Time on GLP-1-RA treatment	Weight loss under GLP-1-RA treatment
Rye et al. [34]	Retrospective	20	RYGB/LSG/VBG/AGB	WR > 10% WL < 20% reached plateau	6.3 years	Liraglutide 3 mg/day	28 weeks	−9.7%
Wharton et al. [35]	Retrospective	117	RYGB/AGB/SG	Received liraglutide 3 mg in the clinic and gained 58% average maximal weight postoperatively	7.8 years	Liraglutide 3 mg/day	7.6 months	−5.5%
Suliman et al. [36]	Retrospective	76	RYGB/SG/Other	Treated in ICLDC clinic and received liraglutide 3 mg	4 years	Liraglutide 3 mg/day	213 days	-6.1%
Abrahamsson et al. [37]	Retrospective	13	GBS	<50% EBWL in 15–20 months	≥2 years	Liraglutide 3 mg/day	15–20 months	−10.4%
Creange et al. [38]	Retrospective	25	LAGB/RYGB/LSG/LAGB and RYGB	Had previous BS and started liraglutide 3 mg	—	Liraglutide 3 mg/day	24 weeks	−9.45%
Rigas et al. [39]	Retrospective	48	LAGB/LSG/GBP	Reached plateau early than expected	—	Liraglutide 3 mg/day	7 months	−13.4%
Talbot et al. [40]	Retrospective	32	Not specified	Gained 15% of weight loss postoperatively	1.1 years	Liraglutide 3 mg/day	9 months	−7.2%
Shehadeh et al. [41]	Retrospective	25	SG/GB/GBP/Last both	Gained >25% of weight loss and did not respond to lifestyle intervention	—	Liraglutide 3 mg/day	3 months	−10%
Muratori et al. [42]	Retrospective	20	LSG/RYGB/LAGB	Gained in BMI postoperative weight loss	4.5 years	Liraglutide 3 mg/day	10.9 months	−5.2 kg/m^2^
Colbourne et al. [43]	Retrospective	68	RYGB/SG/AGB/revisional surgery	IWL after BS: >25% after LSG/RYGB or >20% after LAGB	>1 year after LSG/RYGB or >2 years after LAGB	Liraglutide 3 mg/day	13 months	−5.3%
Lautenbach et al. [45]	Retrospective	44	RYGB/SG	Those with continuous WR after nadir of IWL (EWL < 50% after BS) without type 2 DM	64.7 months	Semaglutide 0.5 mg/week	6 months	−10.3%
Jensen et al. [46]	Retrospective	50	RYGB/SG	Those with WR after BS	72 months	29: Liraglutide 3 mg/day 21: Semaglutide 1 mg/week	6 months	−8.8%
Murvelashvili et al. [47]	Retrospective	207	RYGB/VSG/AGB	Those were prescribed medication after BS due to BMI > 30 kg/m^2^ or >27 kg/m^2^ with obesity related comorbidities	8 years	92: Liraglutide 3 mg/day 115: Semaglutide 1 mg/week	12 months	−12.92% by semaglutide −8.77% by liraglutide
Mok et al. [49]	Prospective, randomized, placebo-controlled	70 35: liraglutide + life style intervention 35: placebo + lifestyle intervention	RYGB/SG	WL < 20% from the day of surgery Suboptimal GLP-1 response (<2× increase in meal stimulated GLP-1 levels)	52.1 months	Liraglutide 3 mg/day vs. placebo saline injection	24 weeks	−8.82% vs. −0.54% Estimated treatment difference: −8.03%

*AGB* adjustable gastric banding, *BMI* body mass index, *BS* bariatric surgery, *DM* diabetes mellitus, *EBWL* estimated body weight loss, *EWL* excess weight loss, *GB* gastric banding, *GBP* gastric bypass, *GLP-1* glucagon-like peptide-1, *GLP-1-RA* glucagon-like peptide-1-receptor agonizt, *ICLDC* imperial college London diabetes center, *IWL* insufficient weight loss, *LAGB* laparoscopic adjustable gastric banding, *LSG* laparoscopic sleeve gastrectomy, *RYGB* Roux-en-Y gastric bypass, *SG* sleeve gastrectomy, *VBG* vertical banded gastroplasty, *VSG* vertical sleeve gastrectomy, *WL* weight loss, *WR* weight regain.

In their retrospective study, Rye et al. [34] reported a median BMI change of 4.7 kg/m^2^ after 28 weeks with liraglutide in patients who regained more than 10% of their total weight loss, lost less than 20% of their weight, or had a plateau of weight loss post MBS. Other retrospective studies showed a 5.5–13.4% weight loss in 3–9 months with liraglutide treatment post MBS [35–44]. In another retrospective analysis of non-diabetic patients receiving 0.5 mg/weekly of semaglutide due to WR or having IWL after BS, Lautenbach et al. [45] reported that 85% of patients had >5% weight loss after 6 months of treatment. However, no significant difference in the rate of weight loss was found between patients with WR as compared to those with IWL [45]. On the other hand, the retrospective observational study by Jensen et al. [46] investigated the effectiveness of liraglutide (3.0 mg/day) and semaglutide (1.0 mg/week) after MBS in patients who regained 15.1% of their total body weight. They showed that 6 months of GLP-1-RA therapy provided 8.8% total weight loss and 2.9 kg/m^2^ BMI loss. Under the GLP-1-RA therapy, patients lost on average two-thirds of the weight regained from their nadir weight. The weight loss in the semaglutide group was found to be significantly higher than in the liraglutide group [46]. Similar results were reported in a recent retrospective study on post-MBS patients who were prescribed semaglutide or liraglutide after 8 years [47]. Significant weight loss was achieved 12 months after the initiation of pharmacotherapy and the weight loss was higher in the semaglutide group than in the liraglutide group [47].

The first randomized, double-blind, placebo-controlled trial using liraglutide after MBS was performed by Lofton et al. [48] and included patients who were 18-120 months after RYGB and had regained ≥10% of total body weight loss after reaching their nadir weight. Liraglutide 3 mg/day resulted in a median weight loss of 9.7%, whereas WR was 1.8% in the placebo group at the end of 56 weeks [48]. In the recently published BARI-OPTIMISE double-blind, placebo-controlled trial, 70 patients post-SG or RYGB who were tested and found to have insufficient GLP-1 response to meal stimulation were randomized to receive liraglutide (3 mg/day) or placebo, in addition to lifestyle interventions [49]. Patients were at least 1 year after their primary MBS and had experienced less than 20% weight loss. The change in body weight was 8.82% after 24 weeks of liraglutide treatment, whereas it was stable in the placebo group. In the liraglutide group, 71.9% of the patients lost more than 5% of their body weight, but only 8.8% of patients in the placebo group lost more than 5% of their weight [49]. The estimated treatment difference was −8.03%, and higher than the weight change difference in the non-bariatric patients treated with liraglutide, which had been reported as −4.8% in a recent systematic review and meta-analysis of randomized controlled trials [50]. Liraglutide and semaglutide have been reported in both retrospective and prospective studies as mostly well-tolerated drugs, with no severe adverse reactions after MBS [44, 46–50].

### GLP-1-RAs and metabolic adaptation

Metabolic adaptation is the difference between the observed resting energy expenditure and the estimated resting energy expenditure post-weight loss [51]. It is a crucial body response to weight loss, with its main purpose being to prevent starvation. In people with obesity populations, metabolic adaptation diminishes resting energy expenditure and results in energy conservation during the process of weight loss; this makes the process of weight loss and weight maintenance more difficult as it progresses. In their study, Wolfe et al. [52] showed that compared with the basal measurements, resting metabolic rate and total daily energy expenditure significantly decreased with weight loss at 6 and 24 months post MBS. They also observed a significant degree of metabolic adaptation at 6 months post MBS [52]. Similarly, Bettini et al. [53] reported a significant reduction in the resting energy expenditure after SG and a significant degree of metabolic adaptation. They also found an inverse association between metabolic adaptation and weight loss after surgery, suggesting that the degree of metabolic adaptation might be one of the factors influencing the extent of weight loss post MBS [53]. In their recent study, Cardia et al. [54] evaluated the correlation of resting metabolic rate per kilogram with weight loss at 6 and 36 months post MBS. They found the rate of achieving a successful weight loss was nearly 3 times higher in post-RYGB patients with resting metabolic rate >2 kcal/kg in comparison with <2 kcal/kg.

In an animal study conducted on rodents, Gabery et al. [55] reported that semaglutide treatment caused weight loss and a lesser decrease in energy expenditure as compared with placebo in calorie-restricted, weight-matched rodents. They also showed that semaglutide treatment actually blunted metabolic adaptation in rodents [55]. Likewise, in preclinical models, GIP-GLP-1 co-agonist has also showed the same phenomena in mice [56]. Very recently, Ravussin et al. [57] showed that subcutaneous tirzepatide at a dose of 15 mg/week decreased body weight by 8–12% in 18 weeks as compared to placebo. Additionally, tirzepatide did not attenuate the reduction of sleeping metabolic rate or 24-h sedentary energy expenditure in comparison to baseline (though not significantly different than placebo), but significantly increased fat oxidation and decreased carbohydrate and protein oxidation as compared to placebo [57]. Hence, further research is needed to elucidate the effects of GLP-1-RAs and GIP-GLP-1 co-agonists on metabolic adaptation in humans post MBS.

### Maintaining “basal-bolus” GLP-1 levels as a treatment approach

**Fig. 1:**
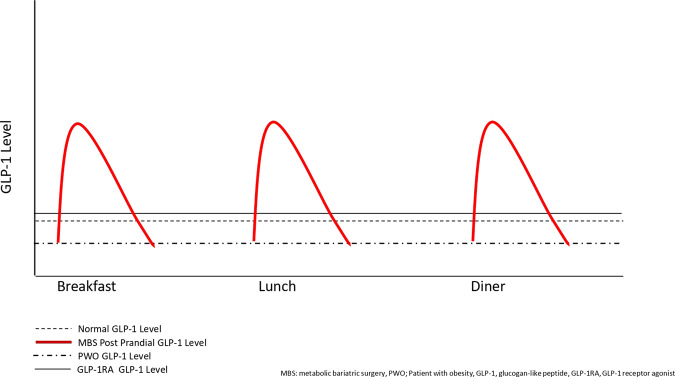
GLP-1 levels and MBS. Hypothetical glucagon-like peptide-1(GLP-1) levels in cases of post metabolic bariatric surgery plus GLP-1 receptor agonists (GLP-1RA).

These studies have clearly shown that MBS corrects post-prandial GLP-1 responses in people with obesity but does not improve fasting GLP-1 levels, which remain lower in some patients as compared to normal-weight individuals. In other words, there is an increased endogenous bolus of GLP-1 after meals post MBS but basal GLP-1 levels remain low. We hypothesize that adding a long-acting GLP-1-RA can increase basal GLP-1 levels and, in combination with native post-prandial GLP-1 bolus restored after MBS, may be considered as a “basal-bolus” treatment for individuals with MBS failure (Fig. 1). Adjunctive basal GLP-1-RA therapy in patients after MBS may be a promising treatment choice in weight management in addition to lifestyle changes, not only due to increased GLP-1 signaling, but also to a putative blunting effect on metabolic adaptation that occurs after MBS, increased fat oxidation, and decreased carbohydrate and protein oxidation. An accurate definition of WR or IWL and monitoring basal or post-prandial GLP-1 levels with a standardized and easily accessible test may be a way to select eligible patients for adjunctive GLP-1-RA therapy post MBS.

In conclusion, WR can occur in surgical and non-surgical patients after a successful process of weight loss. Patients should undergo a comprehensive evaluation to identify the etiology of WR, after which a targeted and personalized intervention should be performed for each patient. Currently available treatments using behavioral and psychosocial interventions lead to modest improvement in outcomes. Pharmacotherapy using GLP-1-RAs in patients after MBS holds promise to be an adjunctive therapy by increasing basal GLP-1 levels while maintaining higher post-prandial GLP-1 levels. Future prospective randomized controlled trials are warranted to understand the efficacy, safety, dosage, and timing of the adjunctive treatment with incretin analogs post MBS.

## Data Availability

Data sharing is not applicable to this article as no datasets were generated or analyzed during the current study.
